# Supplementary material for: Small Bowel Detection for Wireless Capsule Endoscopy Using Convolutional Neural Networks with Temporal Filtering
Source: Diagnostics (Basel). 2022 Jul 31;12(8):1858. doi: 10.3390/diagnostics12081858 (PMC9406835; doi:10.3390/diagnostics12081858)
Supplement: Supplementary file 1 [file diagnostics-12-01858-s001.zip › diagnostics-1817094-supplementary.pdf]

Supplementary Table S1

Case	Small bowel transition				Colon transition			
	Label (frame)	Predict (frame)	Frame error	Time error (sec)	Label (frame)	Predict (frame)	Frame error	Time error (sec)
case 01	5736	5631	105	17.50	72911	72987	76	12.67
case 02	25777	25664	113	37.67	75552	75658	106	35.33
case 03	3409	3306	103	34.33	32088	32114	26	8.67
case 04	22572	22473	99	33.00	45367	45467	100	33.33
case 05	3234	3148	86	28.67	58745	58846	101	33.67
case 06	18493	18440	53	17.67	60816	60872	56	18.67
case 07	2837	2873	36	12.00	50549	50663	114	38.00
case 08	2817	2722	95	31.67	50112	50179	67	22.33
case 09	3517	3400	117	39.00	89046	89157	111	37.00
case 10	542	536	6	2.00	67086	67179	93	31.00
case 11	6850	6737	113	37.67	55683	55613	70	23.33
case 12	2533	2441	92	30.67	91040	91117	77	25.67
case 13	1153	970	183	61.00	49088	49298	210	70.00
case 14	3574	3564	10	3.33	73022	73166	144	48.00
case 15	8958	8855	103	34.33	79408	79518	110	36.67
case 16	2881	2873	8	2.67	50545	50663	118	39.33
case 17	16327	16241	86	28.67	63583	63642	59	19.67
case 18	775	1018	243	81.00	80906	81020	114	38.00
case 19	2537	2441	96	32.00	87520	87588	68	22.67
case 20	10560	10416	144	48.00	80501	80576	75	25.00
case 21	10168	10058	110	36.67	50638	50693	55	18.33
case 22	2947	2817	130	43.33	66815	66937	122	40.67
case 23	50601	50504	97	32.33	153049	153149	100	33.33
case 24	2746	2636	110	36.67	23018	23075	57	19.00
case 25	16622	16512	110	36.67	63618	63647	29	9.67
case 26	1329	1240	89	29.67	45728	45844	116	38.67
case 27	1729	1641	88	29.33	43934	44054	120	40.00
case 28	9301	8973	328	109.33	81905	82029	124	41.33

case 29	6336	6203	133	44.33	62577	62720	143	47.67
case 30	67931	67683	248	82.67	251661	251820	159	53.00
case 31	5305	5211	94	31.33	27931	28036	105	35.00
case 32	9599	9523	76	25.33	43117	43226	109	36.33
case 33	10892	10540	352	117.33	66657	66916	259	86.33
case 34	7057	6932	125	41.67	65686	65714	28	9.33
case 35	2750	2665	85	28.33	31019	31030	11	3.67
case 36	2060	1949	111	37.00	52245	52329	84	28.00
case 37	1286	986	300	100.00	41871	42138	267	89.00
case 38	3684	3650	34	11.33	70241	70263	22	7.33
case 39	10661	10566	95	31.67	31590	31590	0	0.00
case 40	3340	3236	104	34.67	31486	31564	78	26.00
Average	-	-	117.75	38.81	-	-	97.08	32.04
